# A logarithmically amortising temperature effect for supervised learning of wheat solar disinfestation of rice weevil *Sitophilus oryzae* (Coleoptera: Curculionidae) using plastic bags

**DOI:** 10.1038/s41598-023-29594-w

**Published:** 2023-02-14

**Authors:** Mohammed M. Abdelsamea, Mohamed Medhat Gaber, Aliyuda Ali, Marios Kyriakou, Shams Fawki

**Affiliations:** 1 School of Computing and Digital Technology, Birmingham City University, Birmingham, B4 7BD UK; 2 Faculty of Computers and Information, Assiut University, Assiut, 71515 Egypt; 3 Faculty of Computer Science and Engineering, Galala University, Suez, 435611 Egypt; 4 Department of Entomology, Faculty of Science, Ain Shams University, Cairo, 11566 Egypt

**Keywords:** Entomology, Computational methods, Scientific data, Computer science, Computational models, Data acquisition, Data mining, Data processing, Machine learning

## Abstract

This work investigates the effectiveness of solar heating using clear polyethylene bags against rice weevil *Sitophilus oryzae* (L.), which is one of the most destructive insect pests against many strategic grains such as wheat. In this paper, we aim at finding the key parameters that affect the control heating system against stored grain insects while ensuring that the wheat grain quality is maintained. We provide a new benchmark dataset, where the experimental and environmental data was collected based on fieldwork during the summer in Canada. We measure the effectiveness of the solution using a novel formula to describe the amortising temperature effect on rice weevil. We adopted different machine learning models to predict the effectiveness of our solution in reaching a lethal heating condition for insect pests, and hence measure the importance of the parameters. The performance of our machine learning models has been validated using a 10-fold cross-validation, showing a high accuracy of 99.5% with 99.01% recall, 100% precision and 99.5% F1-Score obtained by the Random Forest model. Our experimental study on machine learning with SHAP values as an eXplainable post-hoc model provides the best environmental conditions and parameters that have a significant effect on the disinfestation of rice weevils. Our findings suggest that there is an optimal medium-sized grain amount when using solar bags for thermal insect disinfestation under high ambient temperatures. Machine learning provides us with a versatile model for predicting the lethal temperatures that are most effective for eliminating stored grain insects inside clear plastic bags. Using this powerful technology, we can gain valuable information on the optimal conditions to eliminate these pests. Our model allows us to predict whether a certain combination of parameters will be effective in the treatment of insects using thermal control. We make our dataset publicly available under a Creative Commons Licence to encourage researchers to use it as a benchmark for their studies.

## Introduction

The global population has been estimated to be about 7.8 billion in 2020 and is expected to reach 9.8 billion in 2050^1–4^. Therefore, increasing global grain production is a key factor to meet world food demands. Food production and consumption are expected to increase by approximately 50–70% in 2050^1–3^. On the other hand, post-harvest loss is responsible for huge damage of the total food production especially in some African countries and causes about 20–40% loss, which is a significant loss compared to low food production in the same countries^2^. Insects are the main cause of grain damage and grain losses are estimated to be 30–90%^2,5^. Efforts to reduce post-harvest losses would have a significant impact and improve food availability and food supply compared to limitations and challenges to increase food production^2,6^.

Rice weevil *Sitophilus oryzae* (L.) (Coleoptera: Curculionidae) is one of the most important insect pests of stored wheat and other cereal grains worldwide^5,7^. It is a primary stored-grain insect that attacks whole grains and causes significant grain losses^7,8^. The feeding activity of *S. oryzae* leads to a secondary infestation of pests, fungal diseases, and grain spoilage^7,8^. The mature rice weevil grows 3 to 4 mm long and the larvae feed internally inside the grain kernel through its escape, creating distinctive tiny, round holes known as weevil damage^9^. The eggs are laid on the grain surface and the incubation period to hatch is 2.62–5.85 days. When food is available, male longevity is 42.63–58.72 days, and female longevity is 60.69–77.23 days^8^. Female fecundity rate is 53.60 eggs/lifetime^7^. Rice weevil growth acceleration occurs in temperatures of 25–30 $^\circ$C and 65–75% Relative humidity (r.h.)^7,8^. Most grain storage ecosystems have temperature and r.h. close to the favourable conditions for rice weevil growth and other insects, which also causes a vast loss of grain^2,10^.

Synthetic insecticides and phosphine fumigants are the most effective method used to control stored grain insects^2,6^. These fumigants have some limitations such as relatively high cost and the ability of insects to develop resistance to these insecticides^2,5^. Moreover, improper use of fumigants by smallholders and farmers could lead to health and environmental hazards^11^. In many developing countries in Africa and Asia, grain production depends mainly on small farmers and smallholders rather than large-scale governmental institutions^6^. In these areas, the grains are stored in warehouses and open granaries with a poor storage system and lack of adequate technologies^6^. Therefore, several non-chemical control methods have been used against stored grain insects, such as airtight (hermetic) storage ozone treatment and thermal control^2,4,12^. Thermal control against stored grain insects has been used in many developed and developing countries. Large-scale heating control depends on the heating of storage buildings, large warehouses, and big silos^13,14^. On the other hand, many simple heating systems have been adopted by small farmers in many developing countries. Solar disinfestation against insect pests has been used successfully against soil, museum, and stored grain insects^14^.

Thermal control depends on increasing the temperatures to 40–60 $^\circ$C, which is lethal limits to most of the stored grain insects^15^. Thermal control is a promising technique without any health hazards or development of insect resistance. Different studies have investigated thermal control against rice weevil^15–17^. A complete adult mortality of rice weevil was achieved after 130, 50, 12, and 4 min at 44, 46, 48, and 50 $^\circ$C, respectively^16^. Other studies reported that rice weevil adults were killed after 4 days, and 40 seconds at 39, and 66 $^\circ$C, respectively^15^. According to Beckett^17^ the lethal time to kill 99.9 of the adult population of rice weevil is 37.36–3.71 hours between 42 and 48 $^\circ$C. In one of the authors’ previous studies, solar disinfestation was used against rice weevil inside plastic bags of wheat grains under field conditions^14^. These plastic bags were able to heat up the grains to lethal insect temperatures of 40–55 $^\circ$C and were able to cause 67.6 ± 30 % mortality of rice weevil adults^14^. In the same study various parameters that could affect the heating capacity of plastic bags to kill 100% of adult insects were investigated, but no thermal model were developed for temperature distribution inside the plastic bags. Therefore, the current work aimed to use Machine learning (ML) algorithms to examine the interaction among various parameters to predict the best conditions lead to a complete suppression of insect population. One of the main factors that were considered to be investigated in the initial study was the grain thickness inside the bags. Since grains are considered a good heat insulator^14,18^, it was hypothesised that the time to heat the grains, especially at the bottom of the bags, will increase as the grain thickness inside the bags increases. Therefore, different grain thicknesses were used.

Machine learning (ML) has been used as an alternative to mathematical and statistical modelling in different fields including the agriculture sector^19,20^. Machine learning is very promising at monitoring and predicting the quality of grain. It has shown great potential in the investigation of new grain storage strategies based on different environmental factors. For example, in^21^ artificial neural networks (ANNs) were used as a predictive model in an Internet of Things (IoT) workflow where the gain quality was monitored to predict the quality of the stored grain and hence its deterioration during the post-harvest stages. In^22^, the quality of stored soybean seeds was predicted under different temperature and packaging conditions using several machine learning models, including ANN, REPTree and M5P decision tree algorithms, Random Forest (RF) and linear regression (LR) models. RF model has outperformed other models in predicting the physiological quality indices of such seeds. Mathematical modelling and multivariate analysis were also used^23^ to evaluate the physicochemical properties of early harvest of soybean stored at different packages and temperatures. A prototype wireless sensor network in IoT platform was designed^24^ for real-time monitoring of intergranular equilibrium moisture content, where neural network algorithms were used to predict the physical, physical quality-chemical and microbiological mass of corn stored in bag silos. Likewise, wireless sensor network prototype, IoT platform, and neural network algorithms were used in^25^ for real-time equilibrium moisture content monitoring and predicting grain quality of corn stored in different conditions in silo and raffia bags. Furthermore, previous attempts have been made to study the thermal behaviour of indoor air in greenhouses, where ML was used to predict the inside environment (e.g., temperatures) of plastic greenhouses or solar heating for soil disinfestation against microbes and other organisms. For example, different machine learning models such as Multiple Linear Regression, Support Vector Machine (SVM), Tree Ensembles, and Gaussian Regression Process were used^19^ to predict indoor air temperatures in a Moroccan greenhouse using the outdoor data, where the Gaussian Regression Process model outperformed other models during the validation and testing stages. In^26^, ANNs and SVM models were used to estimate three variables: inside air, soil and plant temperatures, and energy exchange in a polyethylene greenhouse in Shahreza city in Iran, showing a better performance by the Radial Bias Function (RBF) as an ANN model compared to other models.

Unlike previous attempts, this paper introduces a supervised machine learning solution to help better understand the thermal behaviour inside plastic bags to maximise the effectiveness of solar heating against stored grain insects. This is done by introducing a new formula to measure the effectiveness of the solution, and hence formulate the problem as a binary classification problem. To the best of our knowledge, the study is the first of its kind to predict lethal heating conditions of insect pests and is the first to model the effectiveness of thermal control (solutions) using machine learning. Moreover, we adopted model explainability to provide the community with the different conditions that can lead to an effective heating solution for grain disinfestation and help in better understanding the effect of thickness on our solution.

## Materials and methods

### Field experiment

**Figure 1:**
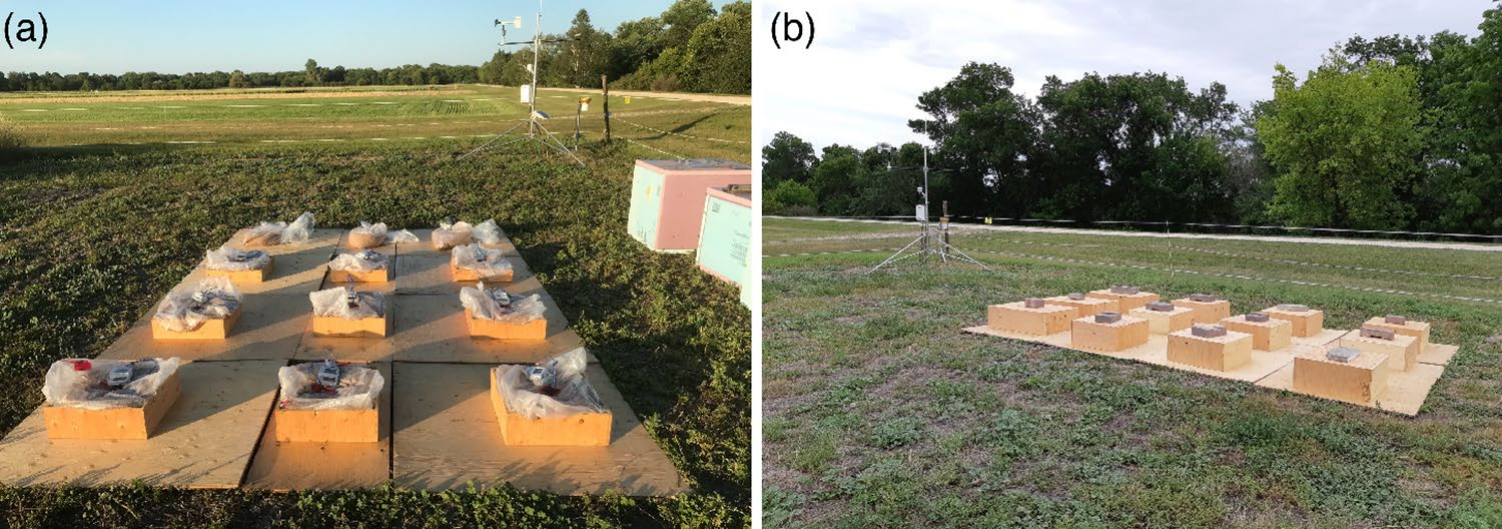
Experimental design: (**a**) Canada 1, after sun exposure the wheat bags were mixed and stacked in the foam boxes. (**b**) Canada 2, during the night all treatments were covered with another wood boxes to prevent any animal disturbance.

In order to investigate the effectiveness of solar heating using clear plastic bags, different parameters have been examined. Some of these variables are solar radiation, air temperature, air humidity, substrate (ground) material, wind speed, grain thickness, and grain moisture content^14,27–29^. It has been also assumed that mixing grain and stacking them inside insulating boxes can help to minimise the time required to heat the grains to temperatures of 40–60 $^\circ$C. Therefore, before the experiment, the wheat grain moisture content was uninformed at 12.5% by a rotating metal container. In the field, wood sheets were also added beneath the plastic bags to uniform the substrate with a low thermal conductivity material. All environmental and temperature data were collected during a field experiment in Canada in the summer of 2018. There were four different treatments of wheat amounts, 16, 21, and 25 kg of wheat inside clear polyethylene bags with wooden boxes and 21 kg of wheat inside a clear polyethylene bag without a wooden box. The Canadian hard red spring wheat *Triticum aestivum* L., (certified seed, SY Slate variety) was used. The wheat was purchased from a grain supplier (Pitura Seeds, Manitoba, Canada) with moisture content of 12.2 ± 0.0%. This work was further subdivided into two parts; the first part was run over 5 days from about 11 am to 8 pm, from 26 to 30 July 2018, where all treatments were kept in the field over the daytime and were mixed and stacked in foam boxes during the night (Canada 1, Fig. 1a). The second part was carried out over 6 days from 7 to 12 of August 2018, where all treatments were kept in the field throughout the day and night without mixing or stacking of the plastic bags (Canada 2, Fig. 1b). The amount of 16, 21, and 25 kg of wheat were representing 9, 13, and 15 cm of wheat thickness, respectively. Temperature fluctuations inside the plastic bags were recorded using thermocouples every 15 min in six positions distributed on the vertical scale in the centre of each bag. These positions were the outer surface of the bag, top of the grain inside the bag, top-middle, middle, middle-bottom, and bottom of the grain. The thermocouples were distributed vertically on equal distance apart along the thickness of each bag. Therefore, wooden boxes were used to uniform the grain thickness, see Fig. 1. Weather data was recorded during the experiment using a weather station at the experimental site. A detailed description of the experimental protocol and how the data were collected is described in the original article and in another data article related to the same work^14,30^.

#### *Dataset*

**Figure 2:**
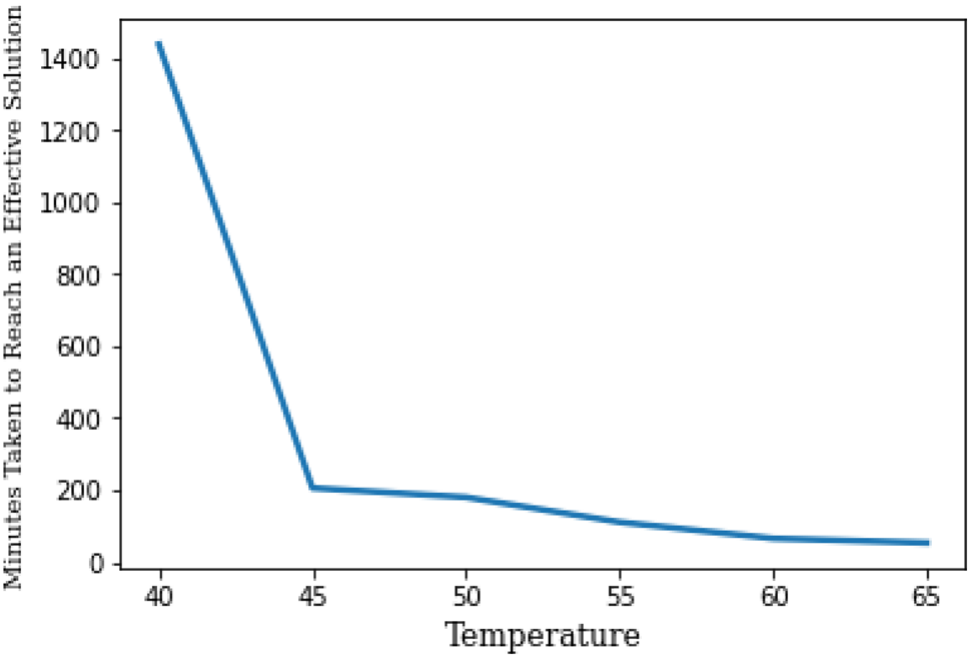
The logarithmic effect of the temperature on the pace of reaching an effective solution.

Our dataset is composed of 7871 observations and 14 features, they are: 16 kg (of wheat inside a wood box), Air Relative Humidity (RH%), Ambient temperatures ($^\circ$C), 21 kg (not in a wood box, binary feature), Solar radiation (M_j_/m^2^), Solar Flux density (K_j_/m^2^), Wind speed (m/s), Mixing and stacking of the grains (binary feature), Air pressure (kpa), 21 kg (of wheat inside a wood box, binary feature), Wind direction (Deg), 25 kg (of wheat inside a wood box, binary feature), Rain (binary feature), and finally the binary Class Label which can be defined by Eq. (1).

1 $$\begin{aligned} {\hat{S}} = \lfloor \sum _{t \ge T} \frac{g_t}{D \times \left( \frac{1}{2^{t-T}}\right) } \rfloor , \end{aligned}$$

where $g_t$ is the time in minutes the solution lasts on a specific temperature *t*, *D* is the time in minutes per day (i.e. $24 \times 60 = 1440$ minutes), and *T* is the temperature threshold to be reached to start a duration of an effective solution to reach lethal heating condition for stored grain insects. For this study, $T = 40$, based on the previous literature mentioned earlier in the introduction section. As per our proposed equation for solution labelling, the higher temperatures will affect the duration of the effective solution logarithmically disproportionally (i.e. it takes a shorter time for an effective solution when the temperature is higher). Figure 2 shows this logarithmic effect. It is worth noting that the equation is applied when the temperature is 40 $^\circ$C or more. Once the temperature drops below 40 $^\circ$C, the value of ${\hat{S}}$ is reset to zero, to be conservative about labelling only certain solutions, and thus modelling the conditions creating them. Finally, the floor function ($\lfloor . \rfloor$) is applied to result in the final value of ${\hat{S}}$, where the value of zero indicates an ineffective solution, and any value $\ge 1$ indicates an effective solution.

### Supervised machine learning models

To examine the dataset and measure the effectiveness of the wheat solar disinfestation solution, we utilised different machine learning models (see Table 1) to predict how effective the solution is in terms of its effect on the rice weevil inside the solar heating bags. The choice of the supervised machine learning methods is based on previous successes reported in the literature utilising them in a variety of applications. Also, we leaned toward a shallow machine learning approach, as explainability is key to the in-hand application, as will be discussed later on.

**Table 1:** A brief description of the machine learning models adopted in this study.

Model	Description	Ensemble	Explainable
Decision Tree (DT)	A decision tree is a statistical machine learning model that consists of nodes and arrows, where each node leads to either a decision or a related consequence. The common ID3 algorithm^31^ has been used in this work as a top-down approach to generate the decision tree. The algorithm splits nodes into sub-nodes using all available features, resulting in the most comparable sub-nodes^31^. Each node represents feature in a category to be classified, and each subset defines a value that the feature can take. A decision tree algorithm can determine which feature is suitable for the split based on entropy and information gain.	NO	YES
Random Forest (RF)	Random Forest Algorithm is constructed based on a collection of classification and Regression Trees (CART) prominent for ensemble learning^32^. Random forest approach is a bagging method that combines deep trees trained on bootstrap samples to achieve a reduced variance output^33^. The class with the highest votes becomes our model’s forecast since each tree in the random forest gives out a class prediction. Bagging includes training a weak learning model on many sets of training data in parallel and then averaging the outcomes of these basic models^34^. RF has demonstrated its robustness in the presence of outliers and the very high dimensional parameter space than other machine learning algorithms.	YES	NO
eXtreme Gradient Boosting (XGB)	Extreme Gradient Boosting or XGBoost is a tree-based machine learning algorithm that works by combining various optimisation techniques to produce outputs within a short time scale^35^. Unlike in Random Forest algorithm where results are obtained by computing the average of all the tree values, in XGBoost, decision trees are built one at a time where results of one tree are computed and summed up for the next tree. Thus, in XGBoost, consideration is given to the gradient of the results. Even though XGBoost computes results faster, the algorithm is complex when compared to other decision tree algorithms.	YES	NO

### Performance evaluation

To evaluate the performance of our machine learning models, we used 10-fold cross-validation and adopted accuracy, recall, precision, and F1-score metrics to quantify the correctly True Positive values, the ratio between the True Positives and the Positives values, and the Harmonic mean of Precision and Recall, respectively^36^. The formulas of the adopted metrics can be defined as:

2 $$\begin{aligned} Precision&=\frac{TP}{TP+FP}\end{aligned}$$

3 $$\begin{aligned} Recall&=\frac{TP}{TP + FN}\end{aligned}$$

4 $$\begin{aligned} F1-score&=\frac{(2 * Precision * Recall)}{(Precision + Recall)} \end{aligned}$$

5 $$\begin{aligned} Accuracy&=\frac{TP+TN}{TP+TN+FP+FN} \end{aligned}$$

where, *TP* is the true positive in the case of the effective solution and *TN* is the true negative in the case of the ineffective solution, while *FP* and *FN* are the incorrect model predictions for non-effective and effective cases.

### Statistical analysis

Time above 40 $^\circ$C and Degree-minutes (DM) inside the plastic bags were estimated. The homogeneity of variances was tested using Levene’s test. Then One-way ANOVA was conducted followed by the Tukey-Kramer HSD. DM model was estimated according to^14,30^ to study the relationship between grain temperatures, exposure time to heat, and rice weevil mortality.

## Results and discussion

### Field experiment

**Figure 3:**
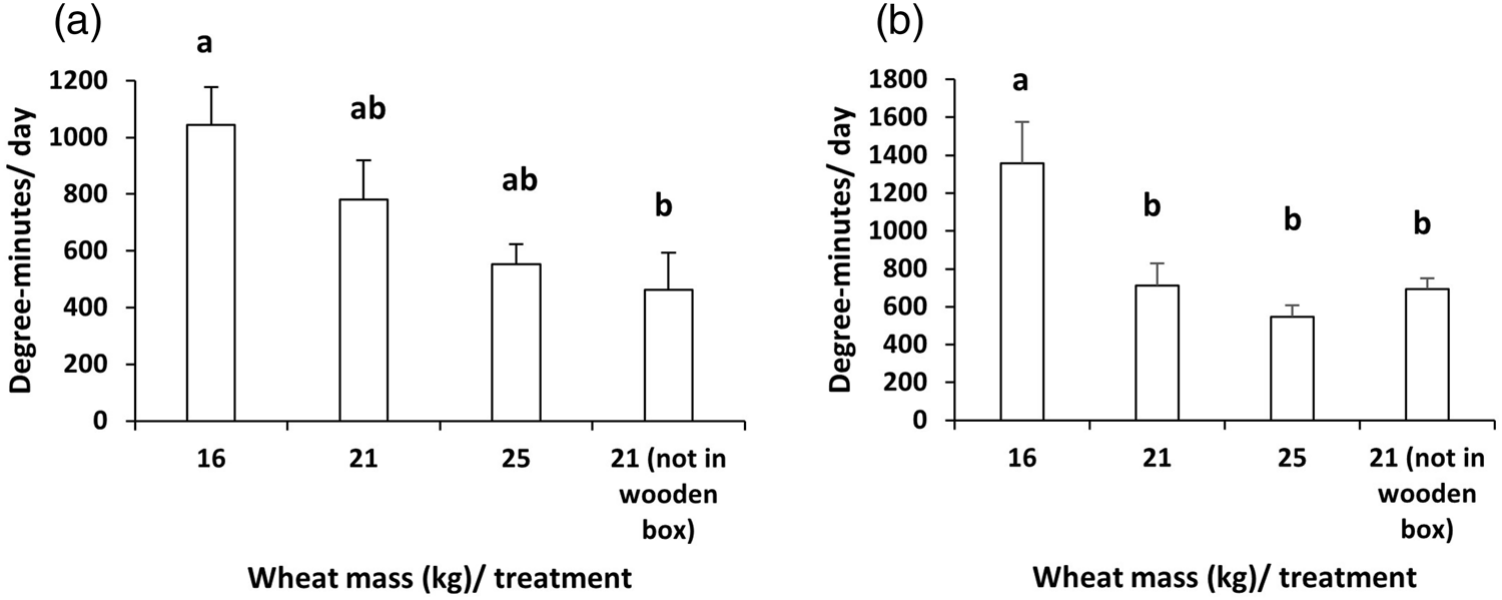
(**a**) Degree-minutes/day ($^\circ$C-min, mean $+$ s.e.m.) of 16, 21, and 25 kg wheat of plastic bags inside wood boxes and another 21 kg of wheat in a plastic bag without a wood box exposed to solar radiation over 5 d in Canada (Canada1). (**b**) Degree-minutes/day ($^\circ$C-min, mean $+$ s.e.m.) of 16, 21, and 25 kg wheat in plastic bags inside wood boxes and another 21 kg of wheat in a plastic bag without a wood box exposed to solar radiation over 6 days in Canada (Canada 2, without stacking). All treatments were kept continuously in the field during the experiment. Different letters indicate significant differences between treatments (*P* < 0.05).

In the first part of the experiment, where the plastic bags of wheat were mixed and stacked (Canada 1), the duration of temperatures that were higher than 40 $^\circ$C (time above 40 $^\circ$C) (min) at the bottom of each plastic bag for each day was significantly higher in 16 kg of wheat compared to other treatments: 2.5 ± 0.6, 0.6 ± 0.2, 0.1 ± 0.1, and 0.1 ± 0.01 (min, mean ± s.e.m.) for 16, 21, 25 kg of wheat inside wood boxes, and 21 kg of wheat without wooden box, respectively (One-way analysis of variance (ANOVA): F$_{3,8}$ = 14.4864, *P* = 0.0013). Time above 40 $^\circ$C at the bottom of 21 and 25 kg of wheat bulks, and 21 kg of wheat without a wooden box was not significantly different from each other. DM were significantly different between different treatments (One-way ANOVA: F$_{3,8}$ = 4.5996, *P* = 0.0375), see Fig. 3a.

In the second part of the experiment, where the plastic bags of wheat were not mixed or stacked (Canada 2), the temperatures only reached 40 $^\circ$C and higher at the bottom of the 16 kg of wheat. None of the other treatments reached 40 $^\circ$C or higher except one replicate out of three replicates in 21 kg of wheat in the wooden box. DM were significantly different between all treatments (One-way ANOVA: F$_{3,7}$ = 8.5934, *P* = 0.0096), see Fig. 3b.

### Machine learning experimental study

Considering that the major challenge in this work is how to handle the imbalanced classes in our dataset as shown in Fig. S1 (see bottom left), four experiments were performed to investigate the performance of different machine learning methods. The first experiment was performed on the original imbalanced dataset and the aim is to evaluate the prediction capability of the different machine learning algorithms on the class imbalance. Results of the first experiment are presented in Table 2.

**Table 2:** Performance evaluation on original dataset.

Algorithm	Accuracy	Recall	Precision	F1-score
Decision Tree (DT)	0.9881	0.5833	0.2100	0.3088
Random Forest (RF)	0.9892	0.6087	0.4200	0.4970
eXtreme Gradient Boosting (XGB)	0.9873	0.0000	0.0000	0.0000

The results shown in Table 2 indicate that the three models achieved an accuracy of not less than 98% in predicting the classes. However, these results are not reliable, as the metrics used are not able to provide further information on which classes are predicted correctly and which are predicted incorrectly. Consequently, the confusion matrices for the three algorithms are calculated and presented in Fig. S1 in the Supplementary Material. As illustrated by Fig. S1, the results of this first experiment show that relying on the accuracy of machine learning methods when coping with an imbalanced dataset might be misleading. To address this challenge, three data resampling techniques were applied, namely, oversampling the minority class, under-sampling the majority class, and the synthetic minority oversampling technique (SMOTE), to the class imbalanced data. The performance of each algorithm on the resampled datasets was evaluated.

The original dataset is transformed by oversampling the minority class technique. In this technique, samples from the minority class are randomly replicated until their number is equal to that of the majority class. Figure S2 (see bottom left) shows the distribution of class labels after applying oversampling the minority class technique. The second experiment was conducted on the oversampled dataset to evaluate the performance of the machine algorithms in classifying both the majority and minority classes. Table 3 presents the results of the second experiment on the oversampled dataset. The results shown in Table 3 indicate that both DT and RF achieved up to 99% accuracy while XGB recorded 95% accuracy. To accurately report the performance of each model, confusion matrices for the three algorithms are computed and presented in Fig. S2 in the Supplementary Material. As demonstrated by Fig. S2, in the case of predicting the minority class, it can be observed that all the three algorithms correctly predicted all samples of the minority class. From these outputs, both DT and RF algorithms have achieved better performance over the XGB algorithm.

**Table 3:** Performance evaluation on oversampled dataset.

Algorithm	Accuracy	Recall	Precision	F1-score
DT	0.9910	0.9823	1.0000	0.9911
RF	0.9910	0.9823	1.0000	0.9911
XGB	0.9591	0.9243	1.0000	0.9607

The original dataset is transformed by applying SMOTE (Synthetic Minority Oversampling Technique)^37^, see the Supplementary Material. This technique is similar to oversampling the minority class technique. However, unlike the oversampling the minority class technique where samples of the minority class are being replicated, in SMOTE, samples of the minority class are synthetically created until their number is equal to that of the majority class. Figure S3 shows the distribution of the class labels after applying the SMOTE technique. The third experiment was performed on the SMOTE dataset to evaluate the performance of the three algorithms. Table 4 presents the results of the fourth experiment. The results presented in Table 4 show that both DT and RF achieved up to 99% accuracy while XGB recorded 95% accuracy. To further evaluate the performance of each algorithm in classifying both majority and minority classes correctly, the confusion matrices for the three algorithms are computed and presented in Fig. S3 in Supplementary Material. Figure S3 shows that XGB performed better in predicting the minority class, followed by RF, then DT. For predicting the majority class, DT performed well, followed by RF, then XGB.

**Table 4:** Performance evaluation on SMOTE dataset.

Algorithm	Accuracy	Recall	Precision	F1-score
DT	0.9946	0.9982	0.9910	0.9946
RF	0.9945	0.9961	0.9929	0.9945
XGB	0.9564	0.9246	0.9940	0.9580

**Figure 4:**
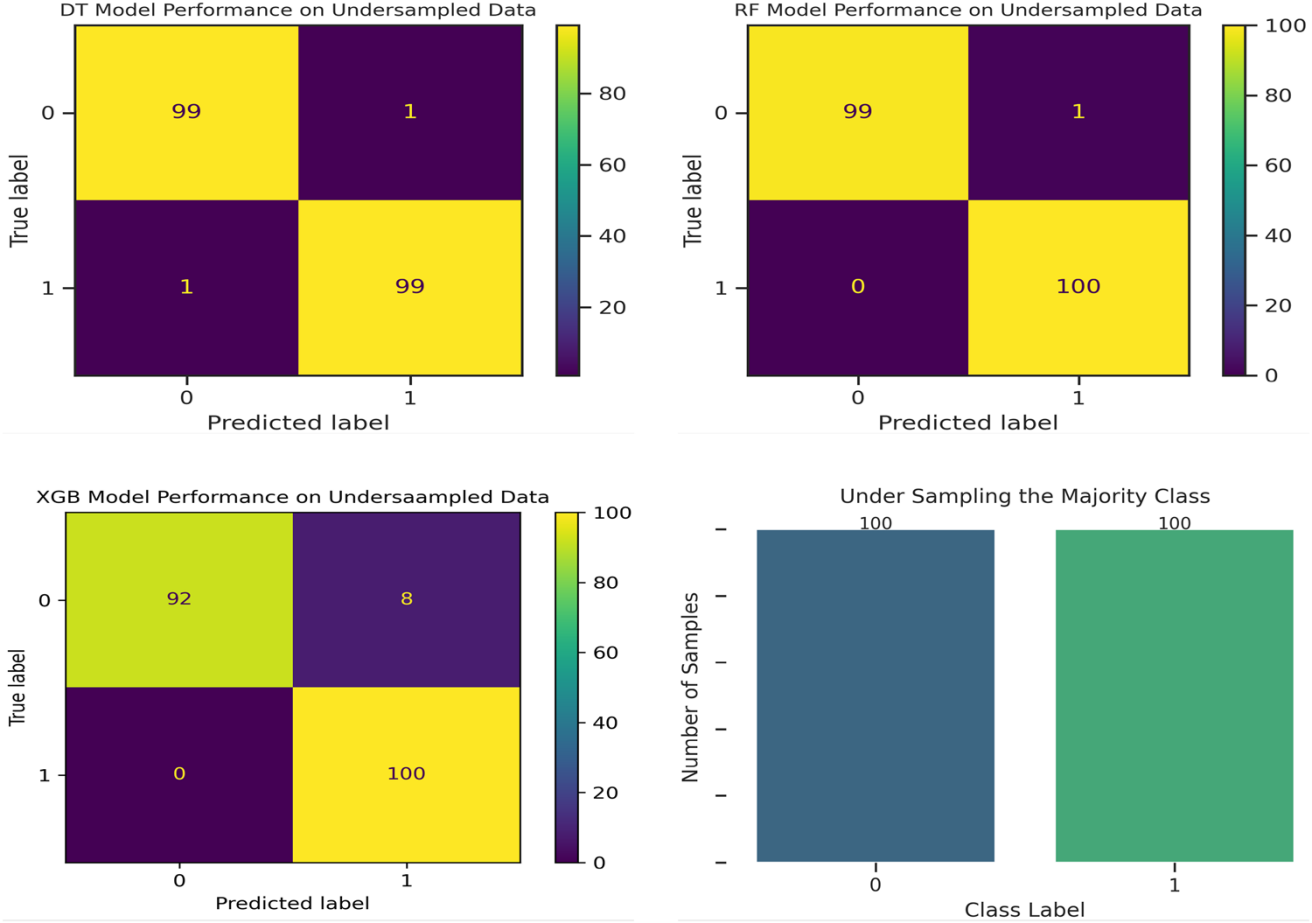
Performance evaluation using confusion matrix on under sampled dataset.

Now, the original dataset is transformed by applying under-sampling the majority class technique. In this technique, samples from the majority class are randomly removed until their number is equal to that of the minority class. Figure 4 (see bottom left) shows the distribution of class labels after applying the under-sampling of the majority class technique. The fourth experiment was conducted on the under-sampled dataset to evaluate the performance of the machine algorithms in classifying both the majority and minority classes. Table 5 presents the results of the third experiment on the under-sampled dataset. The results shown in Table 5 indicate that both DT and RF achieved up to 99% accuracy while XGB recorded 96% accuracy. To accurately demonstrate the performance of each algorithm (in classifying both the majority and minority classes) on the under-sampled dataset, confusion matrices for the three algorithms are computed and presented in Fig. 4.

**Table 5:** Performance evaluation on under sampled dataset.

Algorithm	Accuracy	Recall	Precision	F1-score
DT	0.9900	0.9900	0.9900	0.9900
RF	0.9950	0.9901	1.0000	0.9950
XGB	0.9600	0.9259	1.0000	0.9615

As illustrated by Fig. 4, out of 100 samples for both the majority and minority classes, DT correctly predicted 99 samples and incorrectly predicted 1 sample for both classes. RF predicted 99 samples of the majority class correctly, 1 sample of the majority incorrectly, and all the 100 samples of the minority correctly. XGB predicted 99 samples of the majority class correctly, 8 samples of the majority class incorrectly, and all the 100 samples of the minority correctly. From these outputs, the RF algorithm achieved better performance over the DT and XGB algorithms.

### Interpretation of RF model using SHAP values

As described in the results, the RF model performed well on the under-sampled dataset. However, it is challenging to explain the predictions of RF out of the box. To mitigate this challenge, we apply the eXplainable Artificial Intelligence (XAI) technique known as SHAP (SHapley Additive exPlanations) to provide an intuitive explanation of the RF model’s inner functioning and increase its transparency. Explanations provided are based on the RF model trained on the under-sampled dataset. SHAP is a game-theoretic technique for explaining the output of the machine learning model^38^. It provides global and local explanations using classic Shapley values from the game theory. In addition to estimating feature importance that focuses on interpreting a model in its entirety (global), the SHAP method provides interpretations of separate predictions of the whole model (local).

#### *Global interpretation*

**Figure 5:**
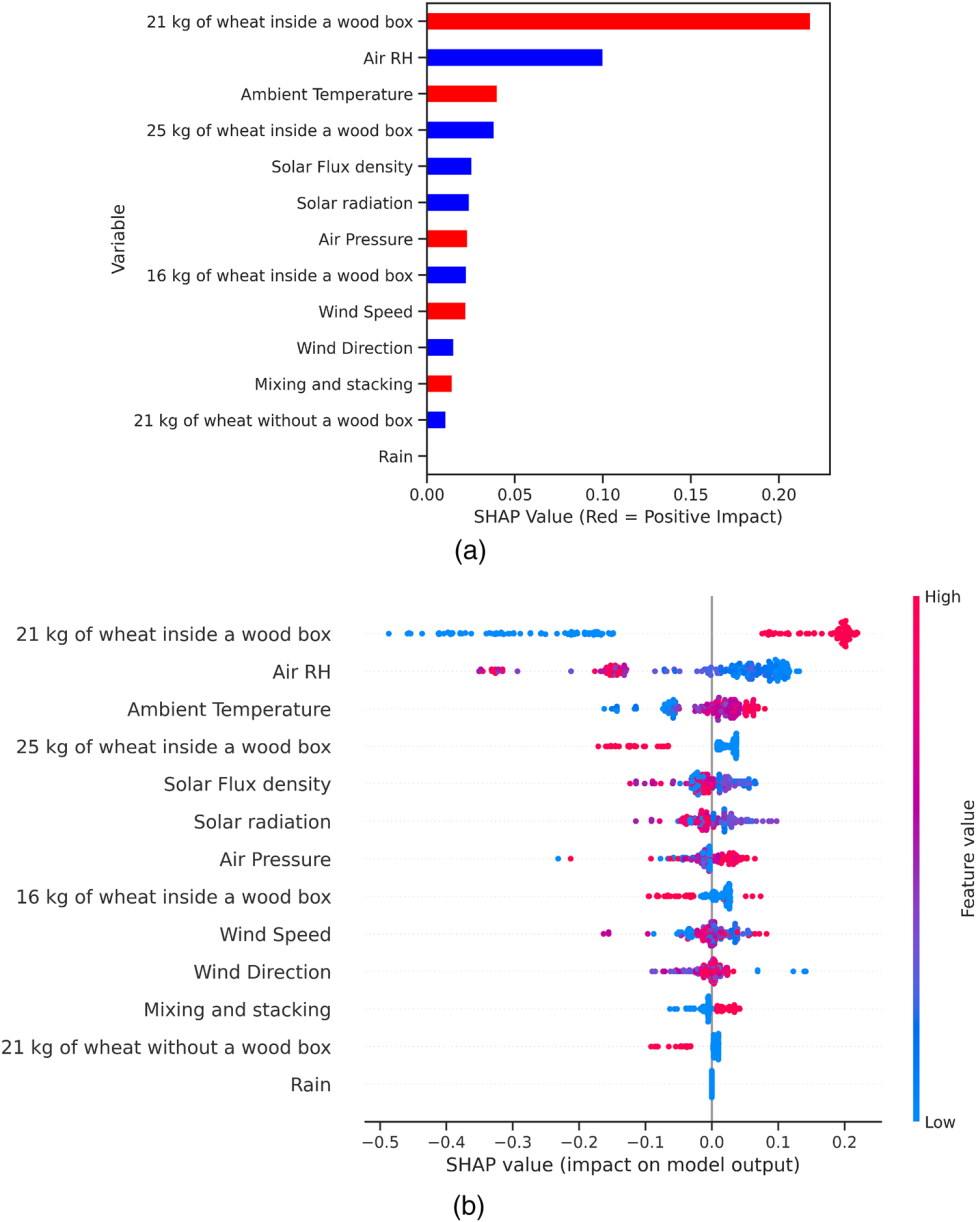
(**a**) Feature importance plot, and (**b**) SHAP summary plot showing feature influence on wheat grains storage solution.

Here, the SHAP values are combined to reveal the contribution of each predictor to the model’s outputs (target variables). The RF model’s feature importance for effective solution of storing wheat grains is shown as a normal bar chart in Fig. 5a and as a SHAP summary plot in Fig. 5b. Figure 5a lists the most influential features in descending order for the efficient solution (class 1). A red bar colour means that the feature has a positive impact on the output, while a blue colour means a negative impact. It can be observed that storing 21 kg of wheat grains inside a wood box has a strong positive impact on output followed by ambient temperature, air pressure, wind speed, and finally mixing and stacking. On the other hand, air RH has a strong negative impact on the output, followed by 25 kg of wheat inside a wood box, solar flux density, solar radiation, 16 kg of wheat inside a wood box, wind direction, then finally 21 kg of wheat without a wood box. Rain has no impact on the output.

The SHAP summary plot shown in Fig. 5b reveals the positive and negative relationships of the features with the target variable. The summary plot reveals the SHAP values of each feature and the dots represent each observation in the dataset. The plot lists all features alongside their importance on the y-axis in descending order of influence and demonstrates whether the effects of their values are associated with a lower or higher prediction. On the x-axis, the position of the dots reveals the impact of the features on the model’s prediction for each observation. For each observation, the colours show whether a feature is low (in blue) or high (in red). Thus, it can be observed that storing 21 kg of wheat grains inside a wood box has a high as well as a positive impact on the model output. The high impact emerges from the red colour, and the positive impact is displayed on the x-axis. Likewise, we will say the feature “Air RH” has a negative impact on the model output.

#### Local interpretation

**Figure 6:**
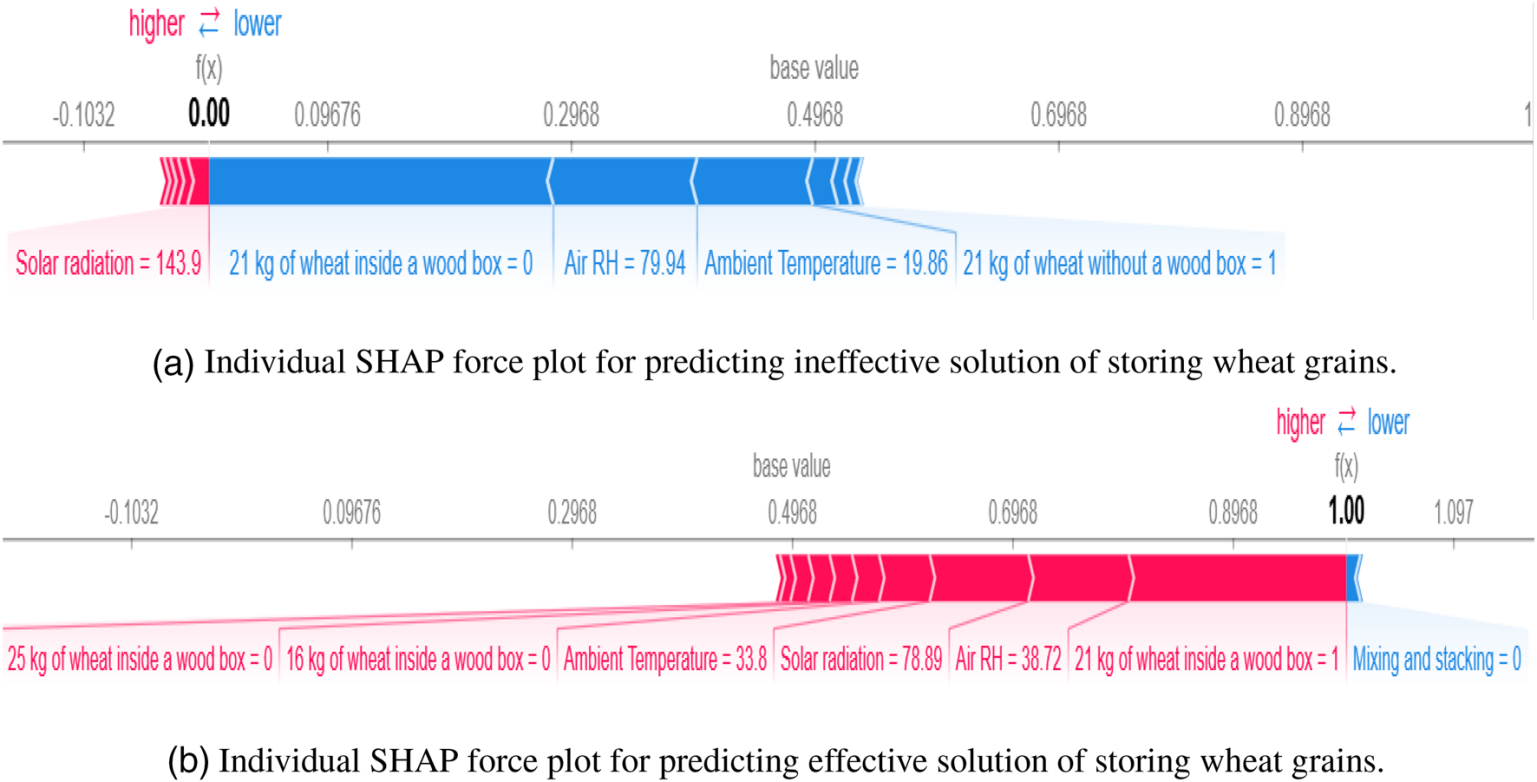
Illustration of SHAP force plots for ineffective (**a**) and effective (**b**) predicted solutions of storing wheat grains.

In Fig. 5a and b, the gradient colours give a picture of the impact each feature has on the solution. To understand how the model arrived at making the decision for each observation, SHAP force charts were created for separate observations. Therefore, each observation or data sample gets its own set of SHAP values which are used to explain why an observation receives its prediction/output and the predictors’ contributions. Figure 6 shows two force plots for separate observations in the dataset. This contains two values: one as a base value and the other as a model prediction. The base value denotes the average model output over the dataset, and the model prediction denotes prediction from the model. These visualisations showed the features that were responsible for the disparity between the model output and the base value. The features that lower the prediction are coloured blue, and those that higher the prediction are coloured red. These plots have the ability to provide recommendations that will inform decision-makers whether or not a particular case will lead to the effective storage of wheat grains.

The RF model was developed based on the default 0.5 threshold of Python’s scikit-learn library where values between 0 and 0.5 belong to class 0 (ineffective solutions) and those between 0.5 and 1 belong to class 1 (effective solutions). For the observation shown in Fig. 6a, the model predicted a value of 0.00 which is lower than the base value of 0.4968 which indicates an ineffective solution for heating the wheat grains. This force plot also shows the features that make the solution ineffective. This solution has solar radiation = 143.9 ($M_j/m^2$). This feature pushes the prediction to higher values as shown in red colour. Even with this force pushing towards higher values, there is a much larger group of features pushing this solution toward lower values as shown in blue colour bars. Each of these features is assigned a value and their combined force causes the model to arrive at this prediction. This simply means that heating of wheat grains would be ineffective if 21 kg of wheat grains is heated without a wood box, at an ambient temperature of 19.86 $^\circ$C, air RH of 79.94%, and solar radiation of 143.9 ($M_j/m^2$). Similarly, the model predicted an effective solution for heating the wheat grains in Fig. 6b. This is shown by the model’s predicted value of 1.00 which is greater than the base value. This plot can also be used to identify which feature attributes make it an effective solution. This solution has a mixing and stacking value of 0 that pushes the prediction toward lowers values as shown in blue colours. Even with this force pushing towards the lower values, there is a much larger group of features pushing this solution toward the higher values as shown in red colour bars. Each of these features is assigned a value and their combined force causes the model to arrive at this prediction. This means that heating wheat grains would be effective if 21 kg of wheat grains are heated inside a wood box, at air RH = 38.72 %, solar radiation = 78.89 ($M_j/m^2$), and ambient temperature of 33.8 $^\circ$C.

The results show that the medium-sized grain amount inside a wood box (21 kg of wheat grains) had a significant effect on our solution to raise temperatures higher than 40 $^\circ$C to kill rice weevil. The heating superiority of the 21 kg box compared to other grain amounts especially the one that has a lesser amount of 16 kg is mainly due to the fact that grains are considered a good insulator^18,39,40^. Recently, puffing grains have been used as a bio-based eco-friendly insulation in building construction^18^. The thicker the amount of grains (insulator), the lower the rate of heat transfer^41^. This means that the thicker the amount of grains, the lower the rate of grain heat gain and the lower the rate of grain heat loss. The optimum grain thickness to achieve the balance between these two processes is a medium amount of grain. Another important feature to optimise our solution is the high ambient temperature. This study was carried out in July and August in Canada, where the maximum air temperature was from 28 to 32 $^\circ$C (Appendices A; Tables A.1 and Table A.2)^42^. The performance of our solar heating bags will be improved in different areas around the world with higher temperature regimes from 30 to 37 $^\circ$C. The summer and autumn seasons are the best time of year to use this heating system, where the daily difference between maximum and minimum air temperatures is the minimum between May and October^41^. The daily rising of air temperature is going faster (about 8 hours) than the dropping from maximum to minimum during the night (about 15 hours)^41^. Therefore, the medium-sized grain amount will perform better than other amounts, especially during the hot seasons. In line with our results, in thermal science, previous studies investigated the effect of material thickness on its thermal isolation^41,43^. These studies reported that there is an optimal thickness for each insulating material.

Finally, mixing and stacking plastic bags had a significant impact on achieving our goal. This effect could be neglected in some cases, when other feature has been met such as high temperature of 33.8 $^\circ$C and using 21 kg of wheat inside wood box. SHAP output is quite similar to the experimental results as mixing and stacking of plastic bags increased the DM of grains, Fig. 3. In the experimental results, DM of 16, 21, and 25 kg of wheat were not significant from each other, Fig. 3a, while according to SHAP output, the medium-sized amount of grains had a positive effect compared to others, Fig. 5a. Finally, the ML model seems to be satisfactory to predict the temperatures inside our closed-heating plastic bags. Similarly, in recent years, different ML model have been developed to used the same environmental data to predict the temperature inside closed greenhouse for planting or soil disinfestation^19,20,26,44^.

## Conclusion

Machine learning is a promising alternative to mathematical and statistical models to study the thermal behaviour inside heating systems used for post harvest thermal control. Machine learning algorithms can effectively examine the interaction among various parameters to predict the effective lethal temperatures for grain insects stored inside clear plastic bags. In this work, we provided a new benchmark dataset, where the experimental and environmental data was collected based on fieldwork during the summer in Canada. We introduced a supervised machine learning solution to help better understand the thermal behaviour inside clear plastic bags to maximise the effective solar heating system against stored grain insects while ensuring that the wheat grain quality is maintained. We utilised the eXplainable Artificial Intelligence (XAI) technique known as SHAP (SHapley Additive exPlanations) to provide an intuitive explanation of the Random Forest model’s inner functioning and increase its transparency. Our main finding is that an optimal medium-sized grain amount inside a wood box (21 kg of wheat grains) had a significant effect on our solution to raise temperatures higher than 40 $^\circ$C against the rice weevil *Sitophilus oryzae* (L.).

## Statement

We confirm that the experimental research and field studies on plants comply with relevant institutional, national, and international guidelines and legislation.

## Supplementary Information

Supplementary Information.

## Data Availability

We have released the dataset under a Creative Commons Attribution 4.0 International License, which can be downloaded at https://sandbox.zenodo.org/record/1067714.

## References

[1] Affognon H, Mutungi C, Sanginga P, Borgemeister C (2015). Unpacking postharvest losses in sub-Saharan Africa: A meta-analysis. World Dev..

[2] Kumar D, Kalita P (2017). Reducing postharvest losses during storage of grain crops to strengthen food security in developing countries. Foods.

[3] Mesterhazy A, Olah J, Popp J (2020). Losses in the grain supply chain: Causes and solutions. Sustainability.

[4] Odokonyero K, Gallo Junior A, Mishra H (2021). Nature-inspired wax-coated jute bags for reducing post-harvest storage losses. Sci. Rep..

[5] Jasrotia P (2022). Nanomaterials for postharvest management of insect pests: Current state and future perspectives. Front. Nanotechnol..

[6] Luo Y, Huang D, Li D, Wu L (2020). On farm storage, storage losses and the effects of loss reduction in china. Resour. Conserv. Recycl..

[7] Rita Devi S, Thomas A, Rebijith KB, Ramamurthy VV (2017). Biology, morphology and molecular characterization of *Sitophilus oryzae* and *S. zeamais* (coleoptera: Curculionidae). J. Stored Prod. Res..

[8] Okram S, Hath TK (2019). Biology of *Sitophilus oryzae* (L.) (coleoptera: Curculionidae) on stored rice grains during different seasons in Terai agro-ecology of West Bengal. Int. J. Curr. Microbiol. Appl. Sci..

[9] Mansoor-ul Hasan AA (2017). Effect of temperature and relative humidity on development of *Sitophilus oryzae* L. (coleoptera: Curculionidae). J. Entomol. Zool. Stud..

[10] Padmasri A (2017). Management of rice weevil (*Sitophilus oryzae* L.) in maize by botanical seed treatments. Int. J. Curr. Microbiol. Appl. Sci..

[11] Rajendran S, Sriranjini V-R (2007). Use of fumigation for managing grain quality. Stewart Postharvest Rev..

[12] Amoah BA, Mahroof RM (2020). Disinfestation of wheat infested with *Sitophilus oryzae* using ozone gas. J. Agric. Urban Entomol..

[13] Mourier H, Poulsen KP (2000). Control of insects and mites in grain using a high temperature/short time (HTST) technique. J. Stored Prod. Res..

[14] Fawki S, Fields PG, Jian F, Yousery A (2022). Control of *Sitophilus oryzae* (coleoptera: Curculionidae) in bags of wheat using solar radiation. J. Stored Prod. Res..

[15] Fields PG (1992). The control of stored-product insects and mites with extreme temperatures. J. Stored Prod. Res..

[16] Yan R, Huang Z, Zhu H, Johnson JA, Wang S (2014). Thermal death kinetics of adult *Sitophilus oryzae* and effects of heating rate on thermotolerance. J. Stored Prod. Res..

[17] Beckett S, Morton R, Darby J (1998). The mortality of *Rhyzopertha dominica* (F.) (coleoptera: Bostrychidae) and *Sitophilus oryzae* (L.) (coleoptera: Curculionidae) at moderate temperatures. J. Stored Prod. Res..

[18] Khoukhi M, Dar Saleh A, Mohammad AF, Hassan A, Abdelbaqi S (2022). Thermal performance and statistical analysis of a new bio-based insulation material produced using grain puffing technique. Constr. Build. Mater..

[19] Allouhi A, Choab N, Hamrani A, Saadeddine S (2021). Machine learning algorithms to assess the thermal behavior of a Moroccan agriculture greenhouse. Clean. Eng. Technol..

[20] Moon T (2019). Interpolation of greenhouse environment data using multilayer perceptron. Comput. Electron. Agric..

[21] Lutz E, Coradi PC (2021). Applications of new technologies for monitoring and predicting grains quality stored: Sensors, internet of things, and artificial intelligence. Measurement.

[22] da Silva Andre G, Coradi PC, Teodoro LPR, Teodoro PE (2022). Predicting the quality of soybean seeds stored in different environments and packaging using machine learning. Sci. Rep..

[23] Lima RE (2021). Mathematical modeling and multivariate analysis applied earliest soybean harvest associated drying and storage conditions and influences on physicochemical grain quality. Sci. Rep..

[24] Coradi PC (2022). Prototype wireless sensor network and internet of things platform for real-time monitoring of intergranular equilibrium moisture content and predict the quality corn stored in silos bags. Expert Syst. Appl..

[25] Lutz É (2022). Real-time equilibrium moisture content monitoring to predict grain quality of corn stored in silo and raffia bags. J. Food Process Eng..

[26] Taki M, Mehdizadeh SA, Rohani A, Rahnama M, Rahmati-Joneidabad M (2018). Applied machine learning in greenhouse simulation; new application and analysis. Inf. Process. Agric..

[27] Al-Mahdouri A, Baneshi M, Gonome H, Okajima J, Maruyama S (2013). Evaluation of optical properties and thermal performances of different greenhouse covering materials. Sol. Energy.

[28] Choab N (2019). Review on greenhouse microclimate and application: Design parameters, thermal modeling and simulation, climate controlling technologies. Sol. Energy.

[29] Pei W, Ming F, Zhang M, Wan X (2023). A thermal conductivity model for insulation materials considering the effect of moisture in cold regions. Cold Reg. Sci. Technol..

[30] Fawki S, Yousery A (2022). Dataset of thermal behaviour and weather data of thermal disinfestation of *Sitophilus oryzae* in plastic bags using solar heating. Data Brief.

[31] Charbuty B, Abdulazeez A (2021). Classification based on decision tree algorithm for machine learning. J. Appl. Sci. Technol. Trends.

[32] Aroef C, Rivan Y, Rustam Z (2020). Comparing random forest and support vector machines for breast cancer classification. Telkomnika.

[33] Bühlmann P (2012). Bagging, boosting and ensemble methods. Handbook of Computational Statistics.

[34] Dietterich TG (2000). An experimental comparison of three methods for constructing ensembles of decision trees: Bagging, boosting, and randomization. Mach. Learn..

[35] Chen, T. & Guestrin, C. Xgboost: A scalable tree boosting system. In *Proceedings of the 22nd ACM SIGKDD International Conference on Knowledge Discovery and Data Mining* 785–794 (2016).

[36] Powers, D. M. Evaluation: From precision, recall and f-measure to roc, informedness, markedness and correlation. arXiv preprint arXiv:2010.16061 (2020).

[37] Jeatrakul, P., Wong, K. W. & Fung, C. C. Classification of imbalanced data by combining the complementary neural network and smote algorithm. In *International Conference on Neural Information Processing* 152–159 (Springer, 2010).

[38] Lundberg, S. M. *et al.* Explainable AI for trees: From local explanations to global understanding. arXiv preprint arXiv:1905.04610 (2019). 10.1038/s42256-019-0138-9 PMC7326367 32607472

[39] Jian F, Jayas DS, White NDG (2013). Specific heat, thermal diffusivity, and bulk density of genetically modified canola with high oil content at different moisture contents, temperatures, and storage times. Trans. ASABE.

[40] Muhammad AA, Akhlaque A, Tasneem A, Muhammad A (2006). Use of solar radiation at village level for thermal disinfestation of wheat stored in galvanized steel bins. Pak. Entomol..

[41] Sahu DK, Sen PK, Sahu GC, Sharma R, Bohidar S (2015). A review on thermal insulation and its optimum thickness to reduce heat loss. Int. J. Innov. Res. Sci. Technol..

[42] Fawki S, Yousery A (2022). Dataset of thermal behaviour and weather data of thermal disinfestation of *Sitophilus oryzae* in plastic bags using solar heating. Data Brief.

[43] Mahlia TM, Taufiq BN, Ismail, Masjuki HH (2007). Correlation between thermal conductivity and the thickness of selected insulation materials for building wall. Energy Build..

[44] D’Emilio, A. *Modeling Soil Thermal Regimes During a Solarization Treatment in Closed Greenhouse by Means of Symbolic Regression via Genetic Programming* 279–286 (Advances in Civil Engineering Materials, 2020).

