# Supplementary material for: A logarithmically amortising temperature effect for supervised learning of wheat solar disinfestation of rice weevil Sitophilus oryzae (Coleoptera: Curculionidae) using plastic bags
Source: Sci Rep. 2023 Feb 14;13:2655. doi: 10.1038/s41598-023-29594-w (PMC9929277; doi:10.1038/s41598-023-29594-w)
Supplement: Supplementary file 1 — Supplementary Information. [file 41598_2023_29594_MOESM1_ESM.pdf]

A Logarithmically Amortising Temperature Effect for Supervised Learning of Wheat Solar Disinfestation of Rice Weevil Sitophilus oryzae (Coleoptera: Curculionidae) using Plastic Bags

Mohammed M. Abdelsamea[1,2,*], Mohamed Medhat Gaber[1,3], Aliyuda Ali[1], Marios Kyriakou[1], and Shams Fawki[4]

[1]School of Computing and Digital Technology Birmingham City University, Birmingham, B4 7BD, UK.
[2]Faculty of Computers and Information, Assiut University, Assiut 71515, Egypt.
[3]Faculty of Computer Science and Engineering, Galala University, Suez 435611, Egypt.
[4]Department of Entomology, Faculty of Science, Ain Shams University, Cairo, 11566, Egypt.
[*]mohammed.abdelsamea@bcu.ac.uk

Synthetic-Minority-Oversampling-Technique (SMOTE)

Synthetic-Minority-Oversampling-Technique (SMOTE) has been adopted here to cope with the class imbalance problem in the dataset. The SMOTE method distributes class distribution equally by generating synthetic data by oversampling the minority class. This is by selecting a minority class as the input vector and identifying its k closest neighbours and selecting one of these neighbours by placing a synthetic point in the line between the point under consideration and its selected neighbour. This process is repeated until the classes of the dataset become evenly distributed.

Supplementary Figures, S1 - S3

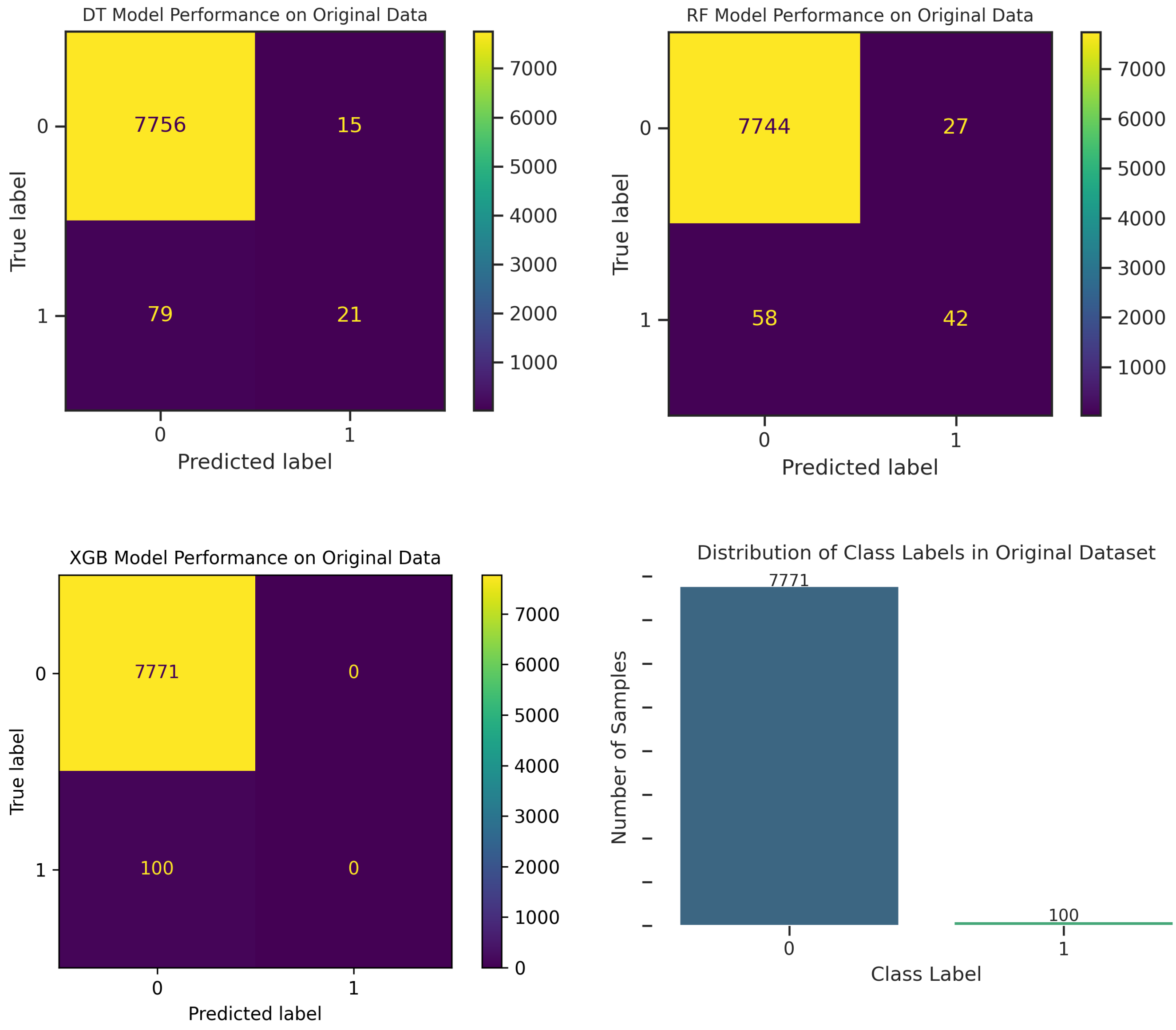

Figure S1. Performance evaluation using confusion matrix on the original dataset. Out of 7771 majority class samples, DT was able to predict 7756 samples correctly while 15 samples were wrongly predicted. Moreover, out of 100 minority class samples, DT was able to predict 21 samples correctly while 79 samples were wrongly predicted. RF algorithm was able to predict 7744 samples of the majority class correctly while 27 were wrongly predicted. In the majority class, RF was able to predict 42 samples correctly while 58 samples were predicted wrongly. XGB algorithm was able to predict all the majority class samples correctly but wrongly predicted all the minority class samples.

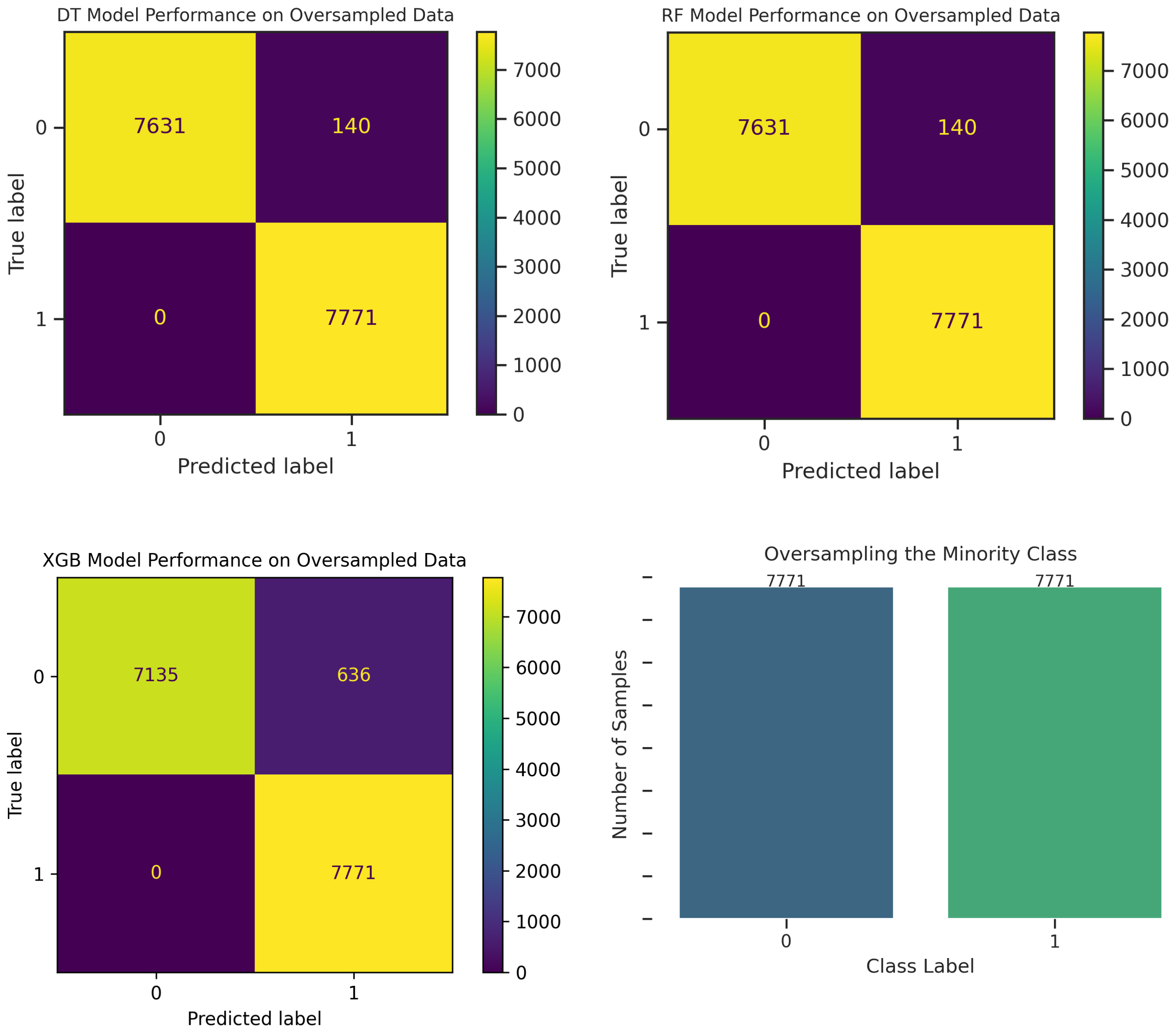

Figure S2. Performance evaluation using confusion matrix on the oversampled dataset. Out of 7771 majority class samples, DT, RF, and XGB were able to correctly predict 7631, 7631, and 7135 samples, respectively, and wrongly predicted 140, 140, and 636 samples, respectively.

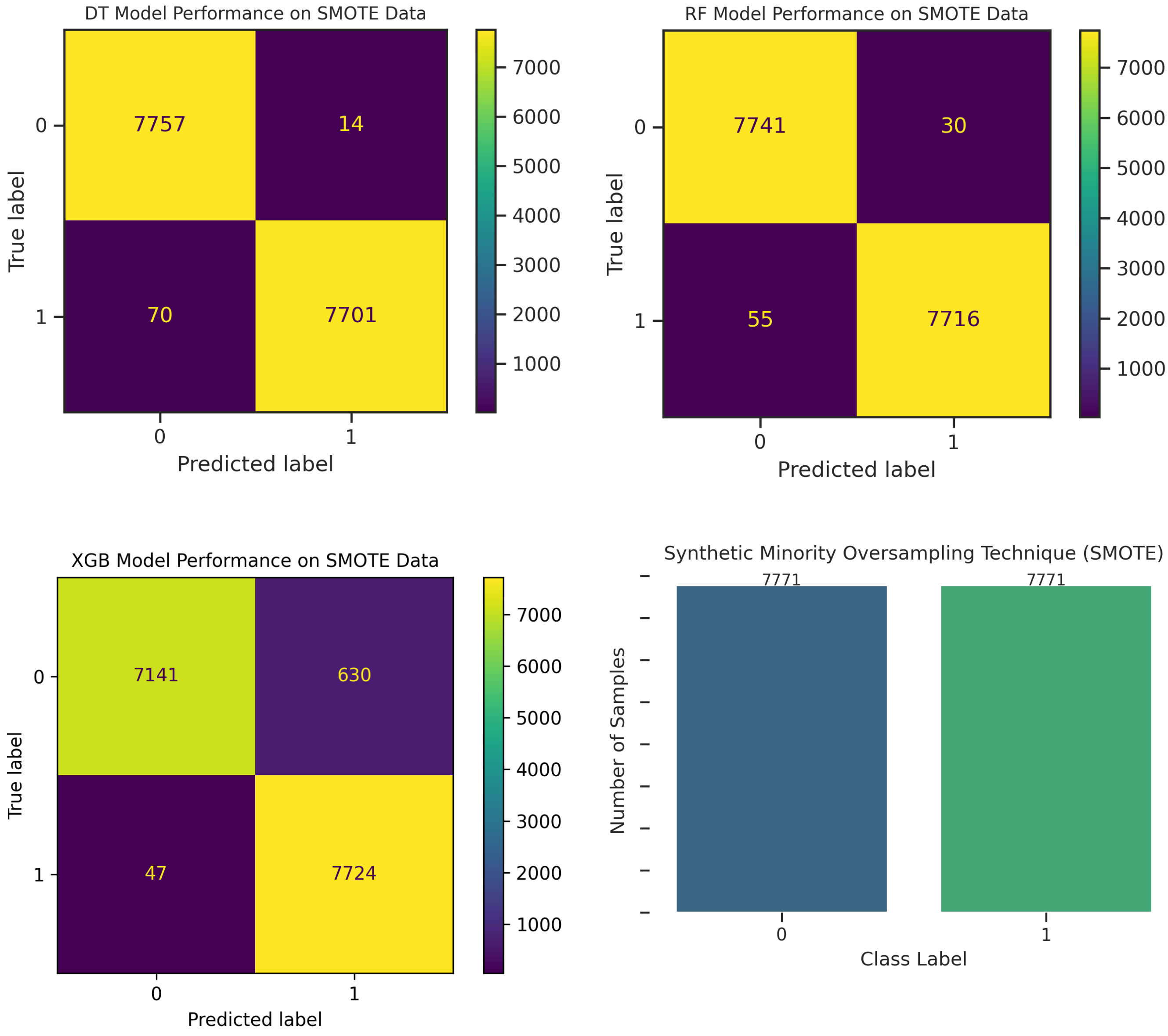

Figure S3. Performance evaluation using confusion matrix on the oversampled dataset using SMOTE method. Out of 7771 samples for both the majority and minority classes, DT correctly predicted 7757 samples and incorrectly predicted 14 samples of the majority class, and correctly predicted 7701 samples and incorrectly predicted 70 samples of the minority class. RF correctly predicted 7741 samples and incorrectly predicted 30 samples of the majority class, correctly predicted 7716 samples, and incorrectly predicted 55 samples of the minority class. XGB correctly predicted 7141 samples and incorrectly predicted 630 samples of the majority class, correctly predicted 7724 samples, and incorrectly predicted 47 samples of the minority class.
